# The impact of COVID-19-pandemic-related adversity on mental health: longitudinal study in Dutch populations with and without mental health disorders

**DOI:** 10.1192/bjo.2023.571

**Published:** 2023-10-10

**Authors:** Patricia Laura Maran, Silvia S. Klokgieters, Erik J. Giltay, Patricia van Oppen, Frederike Jörg, Merijn Eikelenboom, Nathaly Rius Ottenheim, Brenda W. J. H. Penninx, Almar A. L. Kok

**Affiliations:** Health, Medical and Neuropsychology Unit, Leiden University, Leiden, The Netherlands; and Department of Psychiatry, Amsterdam UMC location Vrije Universiteit Amsterdam, Boelelaan, Amsterdam, The Netherlands; Department of Psychiatry, Amsterdam UMC location Vrije Universiteit Amsterdam, Boelelaan, Amsterdam, The Netherlands; Department of Psychiatry, Leiden University Medical Center, Leiden, The Netherlands; Department of Psychiatry, Amsterdam UMC location Vrije Universiteit Amsterdam, Boelelaan, Amsterdam, The Netherlands; and Amsterdam Public Health, Mental Health Programme, Amsterdam, The Netherlands; University Medical Center Groningen, University Center for Psychiatry, Interdisciplinary Centre for Psychopathology and Emotion Regulation, University of Groningen, Groningen, The Netherlands; Research Department, GGZ Friesland, Leeuwarden, The Netherlands; Amsterdam Public Health, Mental Health Programme, Amsterdam, The Netherlands

**Keywords:** COVID-19 pandemic, adversity, depression, anxiety, loneliness

## Abstract

**Background:**

Despite growing concerns about mental health during the COVID-19 pandemic, particularly in people with pre-existing mental health disorders, research has shown that symptoms of depression and anxiety were generally quite stable, with modest changes in certain subgroups. However, individual differences in cumulative exposure to COVID-19 stressors have not been yet considered.

**Aims:**

We aimed to quantify and investigate the impact of individual-level cumulative exposure to COVID-19-pandemic-related adversity on changes in depressive and anxiety symptoms and loneliness. In addition, we examined whether the impact differed among individuals with various levels of pre-pandemic chronicity of mental health disorders.

**Method:**

Between April 2020 and July 2021, 15 successive online questionnaires were distributed among three psychiatric case–control cohorts that started in the 2000s (*N* = 1377). Outcomes included depressive and anxiety symptoms and loneliness. We developed a COVID-19 Adversity Index (CAI) summarising up to 15 repeated measures of COVID-19-pandemic-related exposures (e.g. exposure to COVID-19 infection, negative economic impact and quarantine). We used linear mixed linear models to estimate the effects of COVID-19-related adversity on mental health and its interaction with pre-pandemic chronicity of mental health disorders and CAI.

**Results:**

Higher CAI scores were positively associated with higher increases in depressive symptoms, anxiety symptoms and loneliness. Associations were not statistically significantly different between groups with and without (chronic) pre-pandemic mental health disorders.

**Conclusions:**

Individual differences in cumulative exposure to COVID-19-pandemic-related adversity are important predictors of mental health, but we found no evidence for higher vulnerability among people with (chronic) pre-pandemic mental health disorders.

Since the coronavirus disease (COVID-19) was declared a pandemic by the World Health Organization, it has represented a grave threat to world health, rapidly crossing borders, with more than 768 million confirmed cases and 6.9 million deaths worldwide as of July 2023.^1^ Growing concerns have been raised regarding the impact of this crisis on mental health.^2^ In the general population, many cross-sectional studies have reported increased rates of anxiety, depression and stress during the COVID-19 pandemic.^3-5^ Yet, longitudinal studies among adults found no or little change in depressive and anxiety symptoms compared with pre-pandemic levels ^6,7^ or observed that the initial phases of the lockdown witnessed a high prevalence of depression and anxiety, followed by a fairly rapid decline.^2^ Similarly, a study including seven cohorts from Denmark, France, The Netherlands, and the UK (pooled *N* = 205 084) concluded that the mental health outcomes were poorer at the beginning of the pandemic but tended to steadily improve through subsequent months.^8^ Among individuals with pre-existing mental health disorders, studies including pre-pandemic data concluded that symptoms of depression and anxiety were generally stable or even decreased, at least in adults,^9–12^ whereas loneliness steadily increased among these psychiatric groups, as well as in the general population, exceeding pre-pandemic levels.^2^

Previous studies have shown that mental health outcomes strongly depend on individual sociodemographic characteristics, such as living alone and having a low income, as well as COVID-19-pandemic-related factors such as virus exposure.^11,13^ Accordingly, a recent umbrella review including 14 meta-analyses reporting on the prevalence of depression during and after a COVID-19 infection concluded that the prevalence of depressive symptoms was higher among COVID-19-infected patients compared with both healthcare workers and the general population during the COVID-19 pandemic.^14^ Similarly, Hoogendijk et al^15^ found that people who experienced several COVID-19-pandemic-related stressors, such as COVID-19 infection, financial problems and restrictions in healthcare, were more likely to experience increased depressive and anxiety symptoms, as well as loneliness. However, although valuable, most studies to date did not include pre-pandemic data and did not examine the impact of individual-level total exposure to COVID-19-pandemic-related adversity over time. Therefore, they may have underappreciated the possibility of substantial heterogeneity among subjects in terms of the degree to which mental health changes vary by cumulative exposure to COVID-19-pandemic-related adversity.

Moreover, few studies have focused on groups with mental health disorders, who may be more vulnerable to the impact of COVID-19-pandemic-related adversity. This hypothesis is in line with the stress–vulnerability model,^16^ which posits that whereas stressors can trigger a crisis in all humans, the strength of the response depends on both the intensity of the elicited stress and the individual's tolerance threshold (i.e. one's vulnerability). It is well established that individuals with chronic mental health disorders are more vulnerable to the detrimental effects of stressful events, partly owing to their less favourable coping strategies.^17^ Accordingly, recent studies suggest that individuals lacking sufficient resilience and coping abilities were more likely to suffer adverse psychological consequences during the COVID-19 pandemic.^18,19^ To date, however, there have been no longitudinal studies examining the cumulative effects of COVID-19-pandemic-related stressors such as infection with and course of COVID-19, quarantine, and changes in daily activities on mental health in people with and without pre-existing mental health disorders.

Therefore, the current study employed data from three large Dutch case–control cohort studies to quantify individual, cumulative exposure to COVID-19-pandemic-related adversity across the first 16 months of the COVID-19 pandemic and related this to changes in depressive and anxiety symptoms and loneliness over the course of the pandemic. Furthermore, we examined whether the effects of COVID-19-pandemic-related adversity on these outcomes differed between individuals with and without pre-existing (chronic) mental health disorders. We hypothesised that: (a) higher levels of COVID-19-pandemic-related adversity would be related to greater increases in depressive and anxiety symptoms and loneliness over the course of the pandemic; and (b) the associations between COVID-19-pandemic-related adversity and mental health outcomes would be stronger in persons with more chronic pre-existing mental health disorders. Finally, as an exploratory analysis, we examined whether the associations between COVID-19-pandemic-related adversity and depressive and anxiety symptoms and loneliness differed among types of mental health disorder.

## Method

### Participants

Participants were recruited from three Dutch prospective cohort studies: the Netherlands Study of Depression and Anxiety (NESDA),^20^ the Netherlands Study of Depression in Older Persons (NESDO) ^21^ and Netherlands Obsessive Compulsive Disorder Association Study (NOCDA).^22^ NESDA is an ongoing longitudinal study investigating the course of depression and anxiety disorders among patients (*N* = 2329), their biological siblings (*N* = 367), and controls without mental health disorders (*N* = 652). Between 2004 and 2007, participants aged 16–85 years were recruited from specialised mental healthcare, primary care and the community. Follow-up measurements took place in 2006–2009, 2008–2011, 2010–2013 and 2014–2016. NESDO is a longitudinal study of depression in older people (aged 60–93 years). Between 2007 and 2010, participants with a primary diagnosis of depressive disorder were recruited from out-patient and in-patient mental healthcare and primary care facilities (*N* = 378). Non-depressed controls without lifetime diagnoses of mental health disorders were recruited from primary care (*N* = 132). Follow-up measurements took place in the years 2008–2012 and 2012–2016. NOCDA is a longitudinal study including individuals aged 18–65 years with a lifetime diagnosis of obsessive–compulsive disorder (*N* = 419). Participants were recruited from mental healthcare institutions between 2004 and 2009. Follow-up measurements took place in the years 2006–2011, 2008–2013 and 2012–2016.

The participants in these cohorts who had previously agreed to be contacted for further research (*N* = 2748) were invited via email to participate in a repeated online questionnaire about the mental health impact of the COVID-19 pandemic, i.e. the ‘Covid-19 study’, which was held every 2 to 8 weeks from 1 April 2020 onwards. All participants provided informed consent online. In the present study, data from 15 measurement waves conducted between April 2020 and July 2021 were used. One thousand and fourteen participants participated in at least one wave (overall response rate 62.4%; NESDA: 64%; NESDO: 67.2%; NOCDA: 48%).

The authors assert that all procedures contributing to this work comply with the ethical standards of the relevant national and institutional committees on human experimentation and with the Helsinki Declaration of 1975, as revised in 2008. All procedures involving human subjects/patients were approved by the Institutional Review Board of the Vrije Universiteit Medical Center, Amsterdam (reference number 2020.166).

### Measures

#### Cumulative exposure to COVID-19-pandemic-related adversity index (CAI)

In order to calculate a cumulative index across the observation period and account for the fact that items to be included in the index partly varied per measurement wave, we divided the total period into three subperiods: April 2020 to August 2020 (‘first subperiod’); September 2020 to November 2020 (‘second subperiod’) and December 2020 to July 2021 (‘third subperiod’). These subperiods were based on peaks in national numbers of COVID-19 infections and lockdown measures. Subsequently, we selected participants for whom a CAI score could be calculated in at least two subperiods (*N* = 1377).

The CAI was based on 15 items, which were categorised into nine types of exposure: (a) infection with and course of COVID-19 (items 1, 2 and 3); (b) living alone (item 4); (c) household member's infection with and course of COVID-19 (items 5, 6 and 7); (d) quarantine (item 9); (e) close contact died from COVID-19 (item 8), (f) changes in daily activities (item 10); (g) being inside for prolonged periods of time (item 11); (h) no outdoor space at home (item 12); and (i) negative financial consequences due to COVID-19 pandemic (items 13, 14 and 15). Although living alone and having no outdoor space at home are not directly related to the COVID-19 pandemic, these situations have been proved to have a special potential negative significance during the pandemic^12^ and were therefore considered ‘exposures’. For a detailed description of the items included, see Supplementary Table 2 available at https://doi.org/10.1192/bjo.2023.571.

Each item received a weighted score depending on the assumed severity of the exposure, which was established by discussion within the research team (Supplementary Table 2). For instance, only experiencing COVID-19-related symptoms without a diagnosis of COVID-19 was assigned one point, whereas being diagnosed with COVID-19 received three points. In addition, in cases of mutual dependencies among items (e.g. diagnosis of COVID-19 and experiencing symptoms), the highest score of these items was selected (in this example, three points for COVID-19 diagnosis; Supplementary Fig. 1).

Next, if multiple waves were available for a given period, for each of the nine types of exposures we used the highest score available. Then, for each subperiod we summed the maximum types of exposure scores into a total subperiod score. Finally, based on these subperiod scores, we constructed three variables expressing the overall exposure since the start of the pandemic, which we ultimately used as time-varying predictors in the statistical models. That is, for period 1, the total score for subperiod 1; for period 2, the sum of the scores for subperiods 1 and 2; and for period 3, the sum of the scores for subperiods 1, 2 and 3. Total scores ranged from 0 to 47, with higher scores indicating higher levels of COVID-19-pandemic-related adversity.

#### Pre-pandemic mental health disorder burden

Following Kok et al,^10^ pre-pandemic mental health burden was based on the chronicity of pre-pandemic mental health disorders. In all cohorts, diagnoses of mental health disorders were obtained from structured interviews at each measurement wave. In NESDA and NESDO, the DSM-IV-based Composite Interview Diagnostic Instrument was used for diagnosis.^23^ In NOCDA, the Structured Clinical Interview for DSM-IV axis I disorders was used for diagnosis.^24^ To harmonise the baseline year among the cohorts, only waves from 2006 onwards were included. The percentage of waves between 2006 and 2016 at which participants had any current (6 month recency) diagnosed psychiatric disorder was calculated. Mental health burden was defined using four chronicity groups: no disorders (no lifetime diagnosis); remitted disorder(s) (participants who had mental disorder(s) at baseline that persistently remitted at the remaining measurements or were in a control group that had only a lifetime disorder (0% chronicity)); low–medium chronicity (1–50% waves with disorders); and high chronicity (51–100% waves with disorders). Mental health disorders included major depressive disorder, dysthymia, general anxiety disorder, panic disorder, social phobia, agoraphobia and obsessive–compulsive disorder.

#### Mental health outcomes

Three validated symptom severity scales were included in pre-pandemic waves and COVID-19 questionnaires. Depressive symptoms were assessed with the 16-item Quick Inventory of Symptoms (QIDS),^25^ which assesses nine domains of depression: sleep, mood, weight, concentration, guilt, suicidal ideation, interest, fatigue and psychomotor changes. Respondents were asked to rate the degree to which they experienced each symptom on a scale ranging from 0 (indicating normal functioning) to 3 (indicating severe impairment). The total score ranged from 0 to 27, with a higher score indicating a more severe level of depression symptomatology. Prior studies report good internal consistency (Cronbach's α = 0.86) and good convergent and discriminant validity.^25,26^ In our sample, the internal consistency was good (α_subperiod 1_ = 0.81; α_subperiod 2_ = 0.83; α_subperiod 3_ = 0.83).

Anxiety symptoms were assessed with the Beck Anxiety Inventory (BAI),^27^ which is a 21-item self-reported inventory. Respondents were asked to indicate how strongly each symptom (e.g. fear of losing control) had bothered them in the past week, on a scale ranging from 0 (not at all) to 3 (severely, I could barely stand it). The total score ranged from 0 to 63. Overall, BAI shows high internal consistency (Cronbach's α = 0.92) and good test–retest reliability.^27^ In our sample, the internal consistency was excellent (α_subperiod 1_ = 0.94; α_subperiod 2_ = 0.94; α_subperiod 3_ = 0.94).

Loneliness was assessed with the abbreviated six-item version of the De Jong Gierveld Loneliness Scale (originally 11 items).^28^ Respondents were asked to indicate to what degree each item (e.g. ‘I experience a general sense of emptiness’) applied to them. Response options were ‘yes’, ‘more or less’ (both scored as 1) and ‘no’ (scored as 0). Overall, the De Jong Gierveld Loneliness Scale shows high reliability and validity.^28^ In our sample, the internal consistency was acceptable (α_subperiod 1_ = 0.77; α_subperiod 2_ = 0.79; α_subperiod 3_ = 0.79).

To align with the CAI being calculated for three subperiods, we used the mean of the observed waves in each period as time-varying outcomes.

#### Covariates

We included age, gender (biological sex: female, male), education (low [elementary school], middle [general secondary education] and high [college or university]), number of measurement waves participated in during each subperiod, pre-pandemic partner status, subperiod (coded 0, 1 or 2) and pre-pandemic mental health scores as covariates. Following Pan et al,^9^ the latter was based on average scores for QIDS, BAI and loneliness across all pre-pandemic waves since 2006.

### Statistical analyses

We conducted statistical analyses with SPSS 26.0. First, we examined descriptive statistics (mean and s.d. or median and interquartile range appropriate) of key variables and compared them across chronicity groups using χ^2^ tests and F-tests (analysis of variance), as appropriate.

We used several linear mixed models. First, to examine crude associations between CAI and changes in mental health and loneliness, we included only CAI scores as the time-varying predictor and adjusted for pre-pandemic depressive and anxiety symptoms and loneliness. Second, we examined models with the addition of chronicity of mental health disorders and adjusted for the covariates mentioned above, pre-pandemic depressive and anxiety symptoms and loneliness. Third, we added interaction effects between chronicity of mental health disorders and CAI to examine whether the effect of CAI on outcomes differed between groups with different chronicity of mental health disorders. Missing data were handled by restricted maximum likelihood estimation.

### Sensitivity analyses

We conducted two sensitivity analyses. First, to examine whether the effects of CAI differed between types of mental health disorder, we repeated the above analyses using each psychiatric disorder and the interaction with CAI as predictors (i.e. major depressive disorder, dysthymia, general anxiety disorder, panic disorder, social phobia, agoraphobia and obsessive–compulsive disorder).

Second, because the results could partly depend on our choices regarding the scoring of the exposures, we estimated all models again using an alternative exposure index where each item received a weighted score based on participants’ perceptions. First, we estimated the effect of each type of exposure on the ‘perceived mental health impact’ scale developed earlier (Pan et al^9^). This scale includes nine items (e.g. ‘Because of this period the quality of my sleep is worse’ and ‘In this period it is hard to concentrate’). Answer categories were from 1 (completely disagree) to 5 (completely agree). We then used the standardised regression coefficients as scores in the CAI.

## Results

### Sample characteristics

Participant characteristics across chronicity groups are shown in Table 1. The final sample consisted of *N* = 1377 participants (mean age = 56.84, s.d. = 13.01, min = 28.50, max = 86.00). Compared with individuals with no lifetime disorder, participants with higher chronicity were on average younger, more likely to be women and to not have a partner and less likely to have a high level of education. Moreover, on average, participants with higher chronicity had higher pre-pandemic self-reported depression, anxiety and loneliness than participants with lower chronicity.

**Table 1:** Participants’ characteristics (*N* = 1377) by chronicity of mental health disorders^a^

	No lifetime disorder *N* = 309	Remitted disorder(s) *N* = 342	Low–medium chronicity *N* = 366	High chronicity *N* = 360	Group differences	
	M (s.d.)/*N* (%)	M (s.d.)/*N* (%)	M (s.d.)/*N* (%)	M (s.d.)/*N* (%)	F/χ^2^	*P*
**Demographics**						
Age	59.5 (14.0)	55.9 (13.0)	56.4 (12.8)	55.8 (12.1)	5.7	<0.001
Gender (%)					14.4	0.002
Female	173 (56.0)	225 (65.8)	253 (69.1)	241 (66.9)		
Male	136 (44.0)	117 (34.2)	113 (30.9)	119 (33.1)		
Partner status (%)					20.8	0.002
With partner	257 (83.2)	258 (75.4)	272 (74.3)	252 (70.3)		
No partner	52 (16.8)	84 (24.6)	94 (25.7)	106 (29.7)		
Level of education (%)					21.5	0.001
Basic	7 (2.3)	7 (2.0)	12 (3.3)	18 (5.0)		
Intermediate	141 (45.6)	195 (57.1)	200 (54.6)	210 (58.3)		
High	161 (52.1)	140 (40.9)	154 (42.1)	132 (36.7)		
Source study (%)					244.5	<0.001
NESDA	280 (90.6)	324 (94.7)	339 (92.6)	267 (74.2)		
NESDO	29 (9.4)	0 (0)	10 (2.7)	18 (5.0)		
NOCDA	n/a	18 (5.3)	17 (4.6)	75 (20.8)		
**Type of disorder at baseline** (%)						
MDD	n/a	n/a	220 (60.1)	247 (68.7)		
Dysthymia	n/a	n/a	55 (15.0)	108 (32.8)		
GAD	n/a	n/a	74 (20.2)	126 (35.0)		
Panic Disorder	n/a	n/a	74 (20.2)	167 (46.4)		
Agoraphobia	n/a	n/a	69 (18.9)	147 (40.8)		
Social phobia	n/a	n/a	99 (27.0)	189 (52.5)		
OCD^b^	n/a	n/a	16 (4.4)	75 (20.8)		
**Pre-pandemic mental health outcomes**^c^						
Depressive symptoms	2.57 (1.9)	4.10 (2.7)	6.30 (3.0)	9.74 (4.5)	295.2	<0.001
Anxiety symptoms	2.51 (2.7)	4.93 (4.4)	8.45 (5.3)	15.74 (9.4)	308.6	<0.001
Loneliness	0.88 (1.2)	1.46 (1.7)	2.22 (2.0)	3.30 (2.1)	91.6	<0.001

a. Percentage of previous waves since 2006 with ‘current’ (6 month) mental disorders.

b. OCD only ascertained in NOCDA.

c. Average scores across pre-pandemic waves since 2006.

GAD, generalised anxiety disorder; M, mean; MDD, major depressive disorder; NESDA, Netherlands Study of Depression and Anxiety; NESDO, Netherlands Study of Depression in Older Persons; NOCDA, Netherlands Obsessive Compulsive Disorder Association Study; OCD, obsessive–compulsive disorder.

### CAI scores by chronicity of mental health disorders

Average CAI scores in each period are shown in Table 2. Group differences were found between chronicity groups in each period [period 1: F(3.14) = 9.01, *P* = <0.001; period 2: F(3,14) = 5.94, *P* = <0.001; period 3: F(3,14) = 4.08, *P* = 0.007], with higher CAI scores for participants with high chronicity (mean = 6.37, s.d. = 4.41) than for participants with no lifetime disorders (mean = 5.27, s.d. = 3.98). Moreover, participants with higher chronicity were on average more likely to live alone, to have worse courses of COVID-19 and to be in quarantine. For a detailed description of CAI scores by subperiod, see Supplementary Table 3.

**Table 2:** Cumulative exposure to COVID-19-pandemic-related adversity by chronicity of disorders^a^

		No lifetime disorder *N* = 309	Remitted disorder(s) *N* = 342	Low–medium chronicity *N* = 366	High chronicity *N* = 360	Group differences	
	*N*	M (s.d.)/*N* (%)	M (s.d.)/*N* (%)	M (s.d.)/*N* (%)	M (s.d.)/*N* (%)	F/χ^2^	*P*
**CAI**							
Period 1	1362	2.18 (1.4)	2.37 (1.4)	2.44 (1.3)	2.72 (1.4)	9.0	<0.001
Periods 1–2	1377	3.54 (2.4)	4.05 (2.7)	3.93 (2.4)	4.36 (2.7)	5.9	<0.001
Periods 1–3	1377	5.27 (4.0)	5.99 (4.4)	5.73 (3.9)	6.37 (4.4)	4.1	0.007
**COVID-19 participant**^b^	1377					31.8	0.001
Mild symptoms and diagnosis		17 (5.5)	20 (5.8)	18 (4.9)	23 (6.4)		
Severe symptoms and diagnosis		7 (2.3)	13 (3.8)	4 (1.1)	10 (2.8)		
Admitted to hospital		0 (0.0)	1 (0.3)	3 (0.8)	0 (0.0)		
**COVID-19 household member**^b^	1377					39.9	<0.001
Mild symptoms and diagnosis		22 (7.1)	27 (7.9)	16 (4.4)	18 (5.0)		
Severe symptoms and diagnosis		7 (2.3)	6 (1.8)	4 (1.1)	8 (2.2)		
Admitted to hospital		0 (0.0)	1 (0.3)	2 (0.5)	3 (0.8)		
**Living alone**	1377	80 (25.9)	92 (26.9)	123 (33.6)	140 (38.9)	17.7	<0.001
**Close contact died of COVID-19**^c^	1203	21 (7.6)	37 (12.3)	37 (11.6)	42 (13.6)	5.8	0.121
**Quarantine**	1377	93 (30.1)	135 (39.5)	120 (32.8)	142 (39.4)	9.9	0.019
**No. changes in daily activities**^d^	1335					11.8	0.460
One		185 (59.9)	216 (63.2)	238 (65.0)	245 (68.1)		
Two		58 (18.8)	61 (17.8)	62 (16.9)	47 (13.1)		
Three		8 (2.6)	8 (2.3)	8 (2.2)	5 (1.4)		
Four		0 (0.0)	1 (0.3)	0 (0.0)	2 (0.6)		
**Hours inside per day**	1377					10.6	0.103
>12 h		88 (28.5)	82 (24.0)	91 (24.9)	81 (22.5)		
>18 h		182 (58.9)	217 (63.5)	235 (64.2)	251 (69.7)		
**No outdoor space at home**^d^	1324	10 (3.2)	5 (1.5)	17 (4.6)	16 (4.4)	6.6	0.084
**Financial consequences**	1372					8.8	0.458
Moderate (<20% income decrease)		42 (13.6)	55 (16.1)	49 (13.5)	65 (18.2)		
Severe (>20% income decrease)		46 (14.9)	47 (13.8)	53 (14.5)	60 (16.7)		

a. Percentage of previous waves since 2006 with ‘current’ (6 month) mental health disorders.

b. Question about severity of COVID-19 infection (i.e. mild symptoms, severe symptoms, admitted to hospital) available only in subperiods 2 and 3.

c. Question available only in subperiods 2 and 3.

d. Question available only in subperiod 1.

CAI, Cumulative Adversity Index; M, mean.

### CAI and mental health

#### Depressive symptoms

The results from the linear mixed model showed that the association between CAI and depressive symptoms was positive, indicating that higher CAI scores were related to a greater increase in depressive symptoms (B = 0.54, 9%% CI: 0.42–0.66, *P* < 0.001). Depressive symptoms were on average higher in participants with higher chronicity of mental health disorders pre-pandemic compared with participants with no lifetime disorders (Table 3, model 1). Model 2 included interaction terms between CAI and chronicity; the results obtained with this model showed that higher CAI scores were associated with a greater increase in depressive symptoms relative to the pre-pandemic level of symptoms, and that this effect was stronger with higher chronicity of mental health disorders, although these differences were not statistically significant.

**Table 3:** Impact of cumulative exposure to COVID-19-pandemic-related adversity (CAI) and chronicity of mental health disorders on mental health outcomes

	B	(95% CI)	*P*	B	(95% CI)	*P*
**Outcome: depressive symptoms**	**Model 1**^a^			**Model 2**^a^		
Intercept	1.76	(1.38 to 2.13)	<0.001	1.75	(1.38 to 2.13)	<0.001
CAI	0.54	(0.42 to 0.66)	<0.001	0.41	(0.18 to 0.64)	0.001
Chronicity (ref, no disorder)						
Remitted disorder(s)	0.07	(−0.24 to 0.38)	0.66	0.08	(−0.23 to 0.39)	0.62
Low–medium chronicity	0.23	(−0.10 to 0.56)	0.17	0.24	(−0.09 to 0.57)	0.15
High chronicity	0.74	(0.34 to 1.14)	0.001	0.72	(0.31 to 1.12)	<0.001
Interactions						
CAI × remitted disorder(s)×				0.15	(−0.15 to 0.45)	0.32
CAI × low–medium chronicity				0.07	(−0.23 to 0.38)	0.64
CAI × high chronicity				0.26	(−0.45 to 0.56)	0.10
Period	−0.40	(−0.56 to −0.24)	<0.001	−0.40	(−0.56 to −0.24)	<0.001
**Outcome: anxiety symptoms**	**Model 3**^a^			**Model 4**^a^		
Intercept	0.59	(−0.09 to 1.26)	0.09	0.57	(−0.10 to 1.25)	0.10
CAI	0.82	(0.59 to 1.05)	<0.001	0.68	(0.23 to 1.13)	0.003
Chronicity (ref, no disorder)						
Remitted disorder(s)	0.28	(−0.31 to 0.87)	0.35	0.30	(−0.29 to 0.88)	0.33
Low–medium chronicity	0.80	(0.19 to 1.41)	0.01	0.81	(0.20 to 1.42)	0.01
High chronicity	1.21	(0.48 to 1.94)	0.001	1.20	(0.47 to 1.93)	0.001
Interactions						
CAI × remitted disorder(s)				0.08	(−0.49 to 0.65)	0.80
CAI × low–medium chronicity				0.16	(−0.42 to 0.75)	0.58
CAI × high chronicity				0.27	(−0.29 to 0.83)	0.35
Period	−0.60	(−0.89 to −0.30)	<0.001	−0.59	(−0.89 to −0.30)	<0.001
**Outcome: loneliness**	**Model 5**^a^			**Model 6**^a^		
Intercept	1.16	(0.97 to 1.34)	<0.001	1.16	(0.98 to 1.35)	<0.001
CAI	0.22	(0.16 to 0.28)	<0.001	0.25	(0.12 to 0.38)	<0.001
Chronicity (ref, no disorder)						
Remitted disorder(s)	0.20	(0.04 to 0.36)	0.02	0.19	(0.03 to 0.35)	0.02
Low–medium chronicity	0.58	(0.42 to 0.73)	<0.001	0.57	(0.41 to 0.73)	<0.001
High chronicity	0.90	(0.73 to 1.08)	<0.001	0.91	(0.74 to 1.09)	<0.001
Interactions						
CAI × remitted disorder(s)				0.00	(−0.16 to 0.16)	0.99
CAI × low–medium chronicity				−0.02	(−0.18 to 0.14)	0.80
CAI × high chronicity				−0.10	(−0.25 to 0.06)	0.22
Period	−0.06	(−0.14 to 0.02)	0.13	−0.06	(−0.14 to 0.02)	0.12

a. Models were adjusted for age, gender, education, partner status, period, number of waves participated in per period, and pre-pandemic depressive symptoms/anxiety symptoms or loneliness.

CAI, Cumulative Adversity Index.

#### Anxiety symptoms

Higher CAI scores were associated with a greater increase in anxiety symptoms over time (B = 0.82, 95% CI: 0.59–1.05, *P* < 0.001). Anxiety symptoms were on average higher in participants with higher chronicity of mental health disorders (Table 3, model 3). The results for model 4 showed that higher CAI scores were associated with an increase in anxiety symptoms and that the effect was stronger, though not significantly, in participants with higher chronicity.

#### Loneliness

Findings were similar for loneliness; higher CAI scores were associated with more loneliness (B = 0.22, 95% CI: 0.16–0.28, *P* < 0.001). Participants in higher chronicity groups also had higher levels of loneliness (Table 3, model 5). The results for model 6 showed that higher CAI was associated with an increase in loneliness compared with pre-pandemic levels; however, the interaction effect estimates were near zero, indicating no difference of this effect across chronicity groups.

### Sensitivity analyses

First, we examined whether there were differences in the effects of CAI on outcomes between different types of mental health disorders. We selected generalised anxiety disorder as the reference category, as this type had the closest estimated coefficient to zero when the estimated coefficients of the interaction terms between CAI and type of disorder were compared. For depressive symptoms and anxiety symptoms, we found no significant interaction effects between type of disorder and CAI. For loneliness, we found that the effect of CAI was weaker for participants with social phobia (B = −0.14, 95% CI: −0.25 to −0.03, *P* = 0.02). However, as we tested 18 different interaction effects, this may have been a statistical chance finding.

Second, using the alternative CAI with items weighted on the basis of their effects on participants’ perceived mental health impact revealed similar results to the main analyses. Higher CAI scores remained associated with a higher number of depressive symptoms (B = 0.71, 95% CI: 0.58–0.83, *P* < 0.001), higher number of anxiety symptoms (B = 1.10, 95% CI: 0.86–1.34, *P* < 0.001) and higher levels of loneliness (B = 0.32, 95% CI: 0.25–0.38, *P* < 0.001). Similar to the results of the main analyses, we found no statistically significant interaction effects between CAI and chronicity of mental health disorders for any of the three outcomes (Supplementary Table 4).

## Discussion

This study quantified individual, cumulative exposure to COVID-19-pandemic-related adversity (CAI) across the first 1.5 years of the COVID-19 pandemic in three Dutch cohorts of individuals with and without depressive, anxiety and obsessive–compulsive disorders. We examined its associations with changes in depressive and anxiety symptoms and loneliness compared with pre-pandemic levels and examined whether these associations differed between individuals with different pre-pandemic chronicity of mental health disorders. Our main findings were that individuals with higher chronicity of mental health disorders on average had higher exposure to pandemic-related adversity. In addition, greater CAI was associated with larger increases in symptoms of depression, anxiety and loneliness during the COVID-19 pandemic. However, we did not find evidence that the associations of CAI with mental health and loneliness were significantly stronger in individuals with higher chronicity of mental health disorders.

In line with our findings, Wang et al^29^ showed that individuals with mental health disorders, such as late-life anxiety and depression, had significantly higher risk and worse clinical outcomes of COVID-19 infection compared with those with no mental health disorder. One possible explanation is that late-life depressive symptoms are significantly associated with several medical comorbidities, including an elevated risk of respiratory conditions.^30,31^ It might also be that individuals with mental health disorders have a higher propensity for detecting a COVID-19 virus infection, simply because they pay more attention to their physical symptoms and are therefore more inclined to seek testing compared with individuals without mental health disorders.^32,33^ Furthermore, the difference in scores may have been due to the negative memory bias often present in individuals with mental health disorders, particularly depressive and anxiety disorders, which implies that negative information is more likely to be recalled and reported than neutral or positive information.^34,35^

In line with our expectations, we found that greater individual CAI was associated with a greater increase in symptoms of depression, anxiety and loneliness during the COVID-19 pandemic. Our study showed that despite very modest average increases in mental health problems during the pandemic,^2,13^ it is imperative to take individual differences in exposure to COVID-19-pandemic-related stressors into account. In addition, our findings echoed those of previous studies which examined several COVID-19-related stressors individually, including job loss, death of someone close to you, and financial problems, and found that individuals who had experienced major COVID-19-pandemic-related stressors were more likely to experience poorer mental health outcomes.^36–39^

We expected that the effects of CAI would be stronger in individuals with higher chronicity of mental health disorders. According to Phillips et al,^40^ individuals with pre-existing mental health disorders are more vulnerable to stress-related events because they need to invest more resources in dealing with the original mental disorders and are less likely to engage in active problem-solving strategies when faced with distressing life events. Yet, although we found that effect estimates pointed in the expected direction (i.e. greater impact of CAI on mental health for individuals with a high pre-pandemic chronicity of mental health disorders), we found no strong evidence to support this hypothesis, as the differences were not statistically significant. Therefore, based on our data, we conclude that there is no significant difference in the impact of exposure to COVID-19-pandemic-related adversity on mental health between individuals with and without pre-pandemic mental health disorders.

Sensitivity analyses also suggested few differences in the impact of CAI between types of disorders. One exception was social phobia, for which CAI seemed to have a weaker effect on loneliness compared with generalised anxiety disorder. The fear of others’ critical judgements in social situations is a core feature of social phobia.^41^ Our self-developed index includes items such as living alone and quarantine. In this regard, these pandemic-stressors that generally negatively affect individuals’ mental health may have naturally benefited individuals with social phobia owing to a lack of exposure to anxiety-provoking situations. This may be especially true for levels of loneliness, as loneliness is most likely to be negatively affected by COVID-19.^10,42^

This study had some limitations. First, we reported data from Dutch samples, which limits cross-cultural generalisability. In low- and middle-income countries, exposure to COVID-19-pandemic-related adversity may be greater, with possible stronger effects on individuals’ mental health. Second, the sample was over 60% female; thus, results should be considered in the context of a predominantly female sample. Third, in our sample, cumulative exposure to COVID-19-pandemic-related adversity scores were relatively low compared with the possible maximum cumulative score of 47, indicating that our sample experienced relatively few stressors. Future research is needed to investigate the effects of COVID-19-pandemic-related adversity on mental health in individuals who experienced more stressors. Fourth, our self-developed index for quantifying individual and cumulative exposure to pandemic-related adversity has not been validated. However, we found similar results when comparing the CAI using self-applied weights with an alternative index using weights derived from the effects of each exposure on perceived mental health impact. This suggest that self-applied weights give a reasonable indication of mental health impact. The self-applied weights have the advantage that they can better be replicated by other researchers than weights derived from regression estimates on perceived mental health impact. Despite these limitations, the present study had several strengths, for instance, the repeated observations in three psychiatric cohorts including longitudinal pre-pandemic data with diagnosis interviews for mental health disorders and a healthy control group; the individual-level quantification of exposure to COVID-19-pandemic-related adversity based on various items; and the use of validated symptom severity scales. Moreover, the current study is valuable as it was among the first to capture individual and cumulative exposure to COVID-19-pandemic-related adversity and relate it to mental health outcomes in individuals with and without pre-existing mental health disorders. Considering that COVID-19-pandemic-related adversity was clearly associated with adverse mental health outcomes, assessing the number of stressors individuals experienced during the pandemic might prove helpful in determining individuals’ likelihood of experiencing depression and anxiety symptoms. Importantly, our study findings indicate a significant link between COVID-19-pandemic-related adversity and mental health outcomes, regardless of the presence of a pre-existing mental health disorder. This observation emphasises the crucial need to provide equitable care and attention to individuals, irrespective of their pre-existing mental health status. Furthermore, it underscores that mental health outcomes are primarily shaped by individual-level factors, such as exposure to COVID-19 stressors, rather than solely by pre-existing mental health conditions. Consequently, targeted interventions aimed at vulnerable populations who have experienced heightened levels of cumulative adversity during the pandemic, including low-income groups and individuals with severe COVID-19 trajectories, assume particular importance. Finally, the CAI developed in this study may prove valuable for online mental health interventions related to COVID-19, as it can identify individuals who would benefit most from such interventions.

In sum, the present study showed that the impact of the COVID-19 pandemic varied among individuals, with differing levels of adversity experienced. Cumulative exposure to COVID-19-pandemic-related adversity was clearly related to higher levels of depressive symptoms, anxiety symptoms and loneliness. In addition, our results showed that individuals with mental health disorders may have been more exposed to more COVID-19-pandemic-related stressors. However, although individuals with more chronic mental health disorders reported more absolute cumulative exposure to COVID-19-pandemic-related adversity, the relative impact of these stressors on their mental health was not necessarily more detrimental to their mental health outcomes.

## Supporting information

Maran et al. supplementary material Maran et al. supplementary material

## Data Availability

According to European law (General Data Protection Regulation), data containing potentially identifying or sensitive patients’ information are restricted. However, for academic researchers, data can be made available on request via the NESDA (nesda@ggzingeest.nl), NESDO (d.rhebergen@ggzcentraal.nl) and NOCDA (p.vanoppen@ggzingeest.nl) data access committees.
