# Supplementary material for: The impact of COVID-19-pandemic-related adversity on mental health: longitudinal study in Dutch populations with and without mental health disorders
Source: BJPsych Open. 2023 Oct 10;9(6):e181. doi: 10.1192/bjo.2023.571 (PMC10594261; doi:10.1192/bjo.2023.571)
Supplement: Maran et al. supplementary material [file S2056472423005719sup001.docx]

**Supplementary Appendices**

**Supplementary Table 1.** Availability of data on Covid-pandemic related adversity per wave

|  |  | **WAVE A:** 01/04-12/04 | **WAVE B:** 16-04-27/04 | **WAVE C:** 30/04-11/05 | **WAVE D:** 14-05-25.05 | **WAVE E:** 11/06-25/06 | **WAVE F:** 09/07-21/07 | **WAVE G:** 11/09-14/09 | **WAVE H:** 23/10-06/11 | **WAVE I:** 19/11-24/11 | **WAVE J:** 21/12-08/01 | **WAVE K:** 18/01-01/02 | **WAVE L:** 23/02-08/03 | **WAVE M:** 06/04-20/04 | **WAVE N:** 25/05-05/06 | **WAVE O:** 05/07-19/07 |
| --- | --- | --- | --- | --- | --- | --- | --- | --- | --- | --- | --- | --- | --- | --- | --- | --- |
|  |  | **PERIOD 1 (7 waves)** | | | | | | | **PERIOD 2 (3 waves)** | | | **PERIOD 3 (5 waves)** | | | | |
| 1 | In the past two weeks, have you coughed, had a fever and/or been short of breath? | x | x | x | x | x | x | x | x | x | x | x | x | . | . | . |
| 2 | Have you been diagnosed with coronavirus (COVID-19) by a doctor? | x | x | x | x | x | x | x | x | x | x | x | x | x | x | x |
| 3 | How did your illness progress? | . | . | . | . | . | . | . | x | x | x | x | x | x | x | x |
| 4 | What is currently your home situation? | X | x | x | x | x | x | x | . | . | . | . | . | . | . | . |
| 5 | Has your roommate/any of your roommates had these symptoms in the past two weeks? | x | x | x | x | x | x | x | x | x | x | x | x | . | x | x |
| 6 | Has one (or more) of your household members been diagnosed with coronavirus (COVID- 19) by a doctor? | x | x | x | x | x | x | x | x | x | x | x | x | x | x | x |
| 7 | How did they develop the disease? | . | . | . | . | . | . | . | x | x | x | x | x | x | x | x |
| 8 | Have you been in home quarantine or isolation due to Coronavirus in the past two weeks? | x | x | x | x | x | x | x | x | x | x | x | x | x | x | x |
| 9 | Did someone close to you die from a coronavirus infection? | . | . | . | . | . | . | . | x | x | x | x | x | x | x | x |
| 10 | Changed daily activities | x | x | x | x | x | x | x | . | . | x | x | x | . | . | x |
| 11 | In the past 2 weeks, how many hours a day on average did you spend at home because of the coronavirus outbreak? | x | x | x | x | x | x | x | x | x | x | x | x | x | x | x |
| 12 | Type of outdoors area at home | x | x | x | x | x | x | x | . | . | . | . | . | . | . | . |
| 13 | This period of the coronavirus has had many adverse economic effects on me | x | x | x | x | x | x | x | x | x | x | x | x | x | x | x |
| 14 | Has your household income been affected by the coronavirus outbreak | . | . | . | . | . | . | . | x | x | x | x | . | x | x | x |
| 15 | What is your current work situation? | . | . | . | . | . | . | . | x | x | x | x | . | x | x | x |

| **Supplementary Table 2.** Weights assigned to Covid 19 pandemic exposures included in the Covid-19 pandemic adversity Index (CAI). | | | | | |
| --- | --- | --- | --- | --- | --- |
| Domain | No. | Item description | Answer categories | Score in each period (when observed at least once in that period) | Data available per period |
| Infection with and course of Covid-19 | 1 | In the past two weeks, have you coughed, had a fever and/or been short of breath? (used only in period 1 where item 3 is not available) | Maximum score | 1 | Period 1 and Period 2 |
|  |  |  | Yes | 1 |  |
|  |  |  | No | 0 |  |
|  | 2 | Have you been diagnosed with coronavirus (Covid-19) by a doctor? | Maximum cumulative score based on this item and the previous item 1 | 3 | All periods |
|  |  |  | Yes, on the basis of a positive lab test | 2 |  |
|  |  |  | Yes, on the basis of symptoms | 2 |  |
|  |  |  | No | 0 |  |
|  | 3 | How did your illness progress? | Maximum cumulative score based on this item and the previous items 1 and 2 | 5 | Period 2 and Period 3 |
|  |  |  | Mild complaints | 0 |  |
|  |  |  | Severe symptoms leading to bed rest and/or attention from a physician | 1 |  |
|  |  |  | Serious complaints leading to hospitalization | 2 |  |
| Living alone | 4 | What is currently your home situation (from sub-period 2 used in conjunction with item 5) | Maximum score | 1 | Period 1 |
|  |  |  | alone | 1 |  |
|  |  |  | with partner | 0 |  |
|  |  |  | with partner and children | 0 |  |
|  |  |  | with non-family roommates | 0 |  |
|  |  |  | in a nursing home | 0 |  |
|  |  |  | other, namely | 0 |  |
| Household member’s infection with and course of Covid-19 | 5 | Has your roommate/any of your roommates had these symptoms in the past two weeks? (used only in Period 1 where item 7 is not available) | Maximum score | 0,5 | Period 1 and Period 2 |
|  |  |  | Routing no roommates (i.e., living alone) | 1 |  |
|  |  |  | Yes partner | 0,5 |  |
|  |  |  | Yes child(ren) | 0,5 |  |
|  |  |  | Yes, other roommate | 0,5 |  |
|  |  |  | No | 0 |  |
|  | 6 | Has one (or more) of your household members been diagnosed with coronavirus (Covid- 19) by a doctor? | maximum cumulative score based on this item and the previous item 5 | 2 | All periods |
|  |  |  | Yes | 1,5 |  |
|  |  |  | No | 0 |  |
|  | 7 | How did they develop the disease? (In case of several infected household members, describe the most severe course) | Maximum cumulative score based on this item and the previous items 5 and 6 | 4 | Period 2 and Period 3 |
|  |  |  | Mild complaints | 0 |  |
|  |  |  | Severe symptoms leading to bed rest and/or attention from a physician | 1 |  |
|  |  |  | Serious complaints leading to hospitalization | 2 |  |
| Quarantine | 9 | Have you been in home quarantine or isolation due to Coronavirus in the past two weeks?  Note: Participant receive 0.5 only if they or a household member had symptoms AND neither they nor their household member were diagnosed with Covid-19. | Maximum cumulative score based on this item and the previous items (1, 2 and 5 and 6) | 1/1.5 | All periods |
|  |  |  | Yes | 0.5 |  |
|  |  |  | No | 0 |  |
| Close contact died from Covid-19 | 8 | Did someone close to you die from a coronavirus infection? | Maximum score | 5 | Period 2 and Period 3 |
|  |  |  | Yes | 5 |  |
|  |  |  | No | 0 |  |
| Changes in daily activities | 10 | Changed daily activities (i.e., worked from home, care for children, care for sick family member, other) | Maximum score | 2 | Period 1 |
|  |  |  | 4 activities | 2 |  |
|  |  |  | 3 activities | 1,5 |  |
|  |  |  | 2 activities | 1 |  |
|  |  |  | 1 activity | 0,5 |  |
|  |  |  | No | 0 |  |
| Being inside for long hours | 11 | In the past 2 weeks, how many hours a day on average did you spend at home because of the coronavirus outbreak? (0 to 24 hours) | Maximum score | 1 | All periods |
|  |  |  | 1rst quartile | 0 |  |
|  |  |  | 2^nd^ quartile | 0 |  |
|  |  |  | 3^rd^ quartile | 0,5 |  |
|  |  |  | 4rd quartile | 1 |  |
| No outdoors space at home | 12 | Type of outdoors area at home | Maximum score | 1 | Period 1 |
|  |  |  | No | 1 |  |
|  |  |  | Yes, balcony | 0 |  |
|  |  |  | Yes, garden | 0 |  |
|  |  |  | Yes, balcony and garden | 0 |  |
| Negative financial consequences due to Covid-19 pandemic  Note: for sub-period 2 and 3 the highest score in item 13, 14, and 15 was considered | 13 | This period of the coronavirus has had many adverse economic effects on me | Maximum score | 2 OR | All periods |
|  |  |  | Very much disagree | 0 |  |
|  |  |  | Disagree | 0 |  |
|  |  |  | Neutral | 0 |  |
|  |  |  | Agree | 1 |  |
|  |  |  | Very much agree | 2 |  |
|  |  |  | Not applicable | 0 |  |
|  | 14 | Has your household income been affected by the coronavirus outbreak? | Maximum score | 2 OR | Period 2 and Period 3 |
|  |  |  | No | 0 |  |
|  |  |  | Yes, it has been reduced by 0-20% due to e.g. reduction in working hours, loss of job or closure of  business | 1 |  |
|  |  |  | Yes it has been reduced by >20% due to e.g. reduction in working hours, loss of job or closure of business | 2 |  |
|  |  |  | Yes, it has increased (e.g. more work hours, more activity) | 0 |  |
|  | 15 | What is your current work situation? | I work mainly from home | 0 | Period 2 and Period 3 |
|  |  |  | I've been sent home with pay | 0.5 |  |
|  |  |  | I've been sent home without pay | 2 |  |
|  |  |  | I continue to work at the usual location (e.g. office, factory, construction site) | 0 |  |
|  |  |  | I work through and visit multiple locations for my work | 0 |  |
|  |  |  | I am forced to take (care) leave or vacation | 2 |  |
| Maximum possible score for each period | | | Period 1 | 11 |  |
|  |  |  | Period 2 | 18 |  |
|  |  |  | Period 3 | 18 |  |
| Maximum theoretical cumulative score across three periods  Note: The cumulative score is the sum of the maximum scores for each period | | |  | *47* |  |


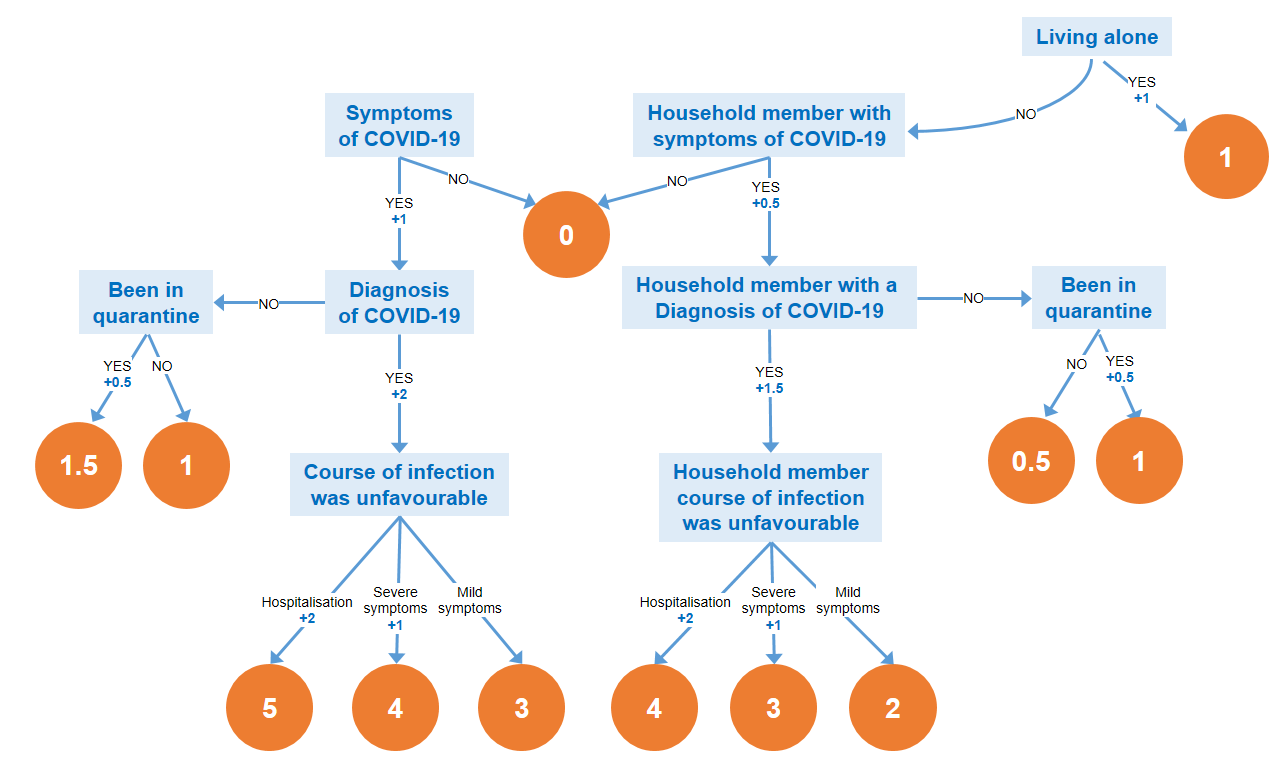
**Supplementary Figure 1.** Scoring of Covid-19 infection and course items included in the Covid-19 pandemic adversity index (CAI)

| **Supplementary Table 3.** Prevalence of exposure to Covid-19 pandemic-related adversity by three sub-period. | | | | | | | | | | | |
| --- | --- | --- | --- | --- | --- | --- | --- | --- | --- | --- | --- |
|  | Sub-period 1 | | | Sub-period 2 | | | Sub-period 3 | | | Group differences | |
|  | *N* | *M* | SD | *N* | *M* | SD | *N* | *M* | SD | F | p |
| Covid-exposure | 1362 | 2.43 | 1.38 | 1166 | 1.86 | 1.81 | 1051 | 2.46 | 2.39 | 39.21 | <.001 |
|  |  | *N* | % |  | *N* | % |  | *N* | % | X^2^ | p |
| Covid-19 infection exposure participant^a^ | 1360 |  |  | 1163 |  |  | 1045 |  |  | 106.61 | <.001 |
| No symptoms nor diagnosis |  | 903 | 66.4 |  | 878 | 75.5 |  | 817 | 78.2 |  |  |
| Symptoms but not diagnosis |  | 431 | 31.7 |  | 244 | 21.0 |  | 130 | 12.4 |  |  |
| (Mild) Symptoms |  | 26 | 1.9 |  | 28 | 2.4 |  | 63 | 6.0 |  |  |
| Severe symptoms |  |  |  |  | 11 | .9 |  | 31 | 3.0 |  |  |
| Hospitalised |  |  |  |  | 2 | .2 |  | 4 | .4 |  |  |
| Covid-19 infection exposure household member | 1361 |  |  | 1162 |  |  | 1045 |  |  | 192.22 | <.001 |
| No symptoms nor diagnosis |  | 685 | 50.3 |  | 684 | 58.9 |  | 599 | 57.3 |  |  |
| Symptoms but not diagnosis |  | 253 | 18.6 |  | 159 | 13.7 |  | 63 | 6.0 |  |  |
| (Mild) Symptoms |  | 14 | 1.0 |  | 33 | 2.8 |  | 70 | 6.7 |  |  |
| Severe symptoms |  |  |  |  | 8 | .7 |  | 23 | 2.2 |  |  |
| Hospitalised |  |  |  |  | 1 | .1 |  | 6 | .6 |  |  |
| Close contact died from Covid-19 |  |  |  | 1159 | 61 | 5.3 | 1044 | 112 | 10.7 | 22.67 | <.001 |
| Quarantine | 1357 | 317 | 23.4 | 1159 | 182 | 15.7 | 1044 | 129 | 13.3 | 46.233 | <.001 |
| Impossibility to go outside (absence of outdoors area at home)^b^ | 1324 | 48 | 3.6 |  |  |  |  |  |  |  |  |
| Economic consequences | 1333 |  |  | 1157 |  | 1018 |  |  |  | 44.341 | <.001 |
| No impact |  | 989 | 74.2 |  | 954 | 82.3 |  | 839 | 82.4 |  |  |
| Mild impact |  | 200 | 15.0 |  | 99 | 8.6 |  | 84 | 8.3 |  |  |
| Severe impact |  | 144 | 10.8 |  | 104 | 9.0 |  | 95 | 9.3 |  |  |
| Time spent inside | 1362 |  |  | 1163 |  |  | 1045 |  |  |  |  |
| More than 16 hours per day |  | 708 | 52.0 |  | 474 | 41.1 |  | 541 | 51.8 | 46.25 | <.001 |
| ^a^ Question about severity of Covid-19 infection (i.e., mild symptoms, severe symptoms, hospitalised) available only in sub-period 2 and 3.  ^b^Question available only in sub-period 1. | | | | | | | | | | | |

| **Supplementary Table 4.** Associations between the alternatively weighted CAI and chronicity of mental health disorders^a^ on mental health outcomes. | | | | | | |
| --- | --- | --- | --- | --- | --- | --- |
| Parameters | b | 95% CI | *p* | b | 95% CI | *p* |
| **Outcome: depressive symptoms** | **Model 1^b^** | | | **Model 2^b^** | | |
| Intercept | 1.95 | 1.32 – 2.59 | <.001 | 1.93 | 1.29 – 2.56 | <.001 |
| CAI | 0.71 | 0.58 – 0.83 | <.001 | 0.68 | 0.44 – 0.93 | <.001 |
| Chronicity (reference = controls) |  |  |  |  |  |  |
| Remitted disorder(s) | 0.03 | -0.28 – 0.34 | .85 | 0.04 | -0.27 – 0.34 | .82 |
| Low-medium chronicity | 0.17 | -0.15 – 0.50 | .30 | 0.19 | -0.14 – 0.52 | .25 |
| High chronicity | 0.65 | 0.25 – 1.05 | .001 | 0.60 | 0.20 – 1.00 | .003 |
| Interactions: |  |  |  |  |  |  |
| CAI x Remitted disorder(s) |  |  |  | -0.01 | -0.32 - 0.29 | .93 |
| CAI x Low-medium chronicity |  |  |  | -0.14 | -0.44 - 0.17 | .39 |
| CAI x High chronicity |  |  |  | 0.28 | -0.03 - 0.60 | .08 |
| Period | -0.50 | -0.65 – -0.34 | <.001 | -0.50 | -0.65 - 0.34 | <.001 |
| **Outcome: anxiety symptoms** | **Model 3^c^** | | | **Model 4^c^** | | |
| Intercept | 0.51 | -0.66 - 1.67 | .40 | 0.47 | -0.70 - 1.63 | .43 |
| CAI | 1.10 | 0.88 - 1.34 | <.001 | 0.85 | 0.38 - 1.33 | <.001 |
| Chronicity (reference = controls) |  |  |  |  |  |  |
| Remitted disorder(s) | 0.26 | -0.33 - 0.84 | .39 | 0.30 | -0.29 - 0.89 | .32 |
| Low-medium chronicity | 0.74 | 0.14 - 1.35 | .02 | 0.77 | 0.16 - 1.38 | .01 |
| High chronicity | 1.14 | 0.42 - 1.87 | <.002 | 1.12 | 0.39 - 1.85 | .003 |
| Interactions: |  |  |  |  |  |  |
| CAI x Remitted disorder(s) |  |  |  | 0.04 | -0.55 - 0.63 | .90 |
| CAI x Low-medium chronicity |  |  |  | 0.31 | -0.29 - 0.90 | .31 |
| CAI x High chronicity |  |  |  | 0.56 | -0.02 - 1.15 | .06 |
| Period | -0.81 | -1.10 – -0.53 | <.001 | -0.81 | -1.10 - -0.52 | <.001 |
| **Outcome: loneliness** | **Model 5^d^** | | | **Model 6^d^** | | |
| Intercept | 1.60 | 1.29 - 1.91 | <.001 | 1.60 | 1.29 - 1.91 | <.001 |
| CAI | 0.32 | 0.25 - 0.38 | <.001 | 0.36 | 0.23 - 0.50 | <.001 |
| Chronicity (reference = controls) |  |  |  |  |  |  |
| Remitted disorder(s) | 0.18 | 0.02 - 0.33 | .04 | 0.17 | 0.01 - 0.33 | .04 |
| Low-medium chronicity | 0.54 | 0.39 - 0.70 | <.001 | 0.54 | 0.38 - 0.69 | <.001 |
| High chronicity | 0.86 | 0.68 - 1.03 | <.001 | 0.86 | 0.68 - 1.03 | <.001 |
| Interactions: |  |  |  |  |  |  |
| CAI x Remitted disorder(s) |  |  |  | -0.07 | -0.23 - 0.09 | .40 |
| CAI x Low-medium chronicity |  |  |  | -0.03 | -0.19 - 0.12 | .67 |
| CAI x High chronicity |  |  |  | -0.07 | -0.23 - 0.09 | .40 |
| Period | -0.13 | -0.21 – -0.06 | <.001 | -0.14 | -0.21 – -0.06 | <.001 |
| ^a^ Percentage of previous waves since 2006 with ‘current’ (6-month) mental health disorders.  ^b^ Model includes age, gender, partner status, period, number of waves participated per period, and pre-pandemic depressive symptoms as covariates.  ^c^ Model includes age, gender, partner status, period, number of waves participated per period, and pre-pandemic anxiety symptoms as covariates.  ^d^ Model includes age, gender, partner status, period, number of waves participated per period, and pre-pandemic loneliness as covariates. | | | | | | |

In the alternative CAI, items received a weighted score based on the estimated association between each of the eight exposures and the perceived mental health impact reported by participants. Following Pan et al. [9], perceived mental health impact on their emotional state and health behaviours was assessed by using nine items (i.e., Because of this period the quality of my sleep is worse). Answer categories were 1 (completely disagree) to 5 (completely agree).
